# Asymmetric adiabatic couplers for fully-integrated broadband quantum-polarization state preparation

**DOI:** 10.1038/s41598-017-17094-7

**Published:** 2017-12-04

**Authors:** Hung-Pin Chung, Kuang-Hsu Huang, Kai Wang, Sung-Lin Yang, Shih-Yuan Yang, Chun-I Sung, Alexander S. Solntsev, Andrey A. Sukhorukov, Dragomir N. Neshev, Yen-Hung Chen

**Affiliations:** 1 Department of Optics and Photonics, National Central University, Jhongli, 32001 Taiwan; 2 Nonlinear Physics Centre, Research School of Physics and Engineering, The Australian National University, Canberra, ACT 2601 Australia; 3 School of Mathematical and Physical Sciences, University of Technology Sydney, Ultimo, NSW 2007 Australia

## Abstract

Spontaneous parametric down-conversion (SPDC) is a widely used method to generate entangled photons, enabling a range of applications from secure communication to tests of quantum physics. Integrating SPDC on a chip provides interferometric stability, allows to reduce a physical footprint, and opens a pathway to true scalability. However, dealing with different photon polarizations and wavelengths on a chip presents a number of challenging problems. In this work, we demonstrate an on-chip polarization beam-splitter based on z-cut titanium-diffused lithium niobate asymmetric adiabatic couplers (AAC) designed for integration with a type-II SPDC source. Our experimental measurements reveal unique polarization beam-splitting regime with the ability to tune the splitting ratios based on wavelength. In particular, we measured a splitting ratio of 17 dB over broadband regions (>60 nm) for both H- and V-polarized lights and a specific 50%/50% splitting ratio for a cross-polarized photon pair from the AAC. The results show that such a system can be used for preparing different quantum polarization-path states that are controllable by changing the phase-matching conditions in the SPDC over a broad band. Furthermore, we propose a fully integrated electro-optically tunable type-II SPDC polarization-path-entangled state preparation circuit on a single lithium niobate photonic chip.

## Introduction

Lithium Niobate (LiNbO_3_) is a ferroelectric material with relatively large nonlinear-optic and electro-optic (EO) coefficients^1^ and has been widely used in numerous applications including the implementation of EO intensity/phase modulators^2^, EO polarization-mode converters (PMCs)^3^, optical wavelength converters such as optical parametric oscillators^4,5^ and quantum light sources^6–8^ that open a range of useful applications of LiNbO_3_ in quantum optics. Besides, LiNbO_3_ is also a popular substrate for constructing low-loss, high-quality optical waveguides, advantageous for realizing those photonic devices with high-efficiency and compatibility. All these attractive features of LiNbO_3_ make it a promising platform for developing integrated quantum photonic circuits; the reviews of several important material platforms including LiNbO_3_ for implementing on-chip photon-pair generators and quantum photonic systems have been made elsewhere^9,10^. In particular, high-brightness polarization entangled photon pairs can be generated via type-II phase-matched spontaneous parametric down-conversion (SPDC) based on quasi-phase matched (QPM) LiNbO_3_ waveguides^6,7^. Such photon pairs from a single waveguide, once generated, typically requires a polarization beam splitting in order to utilize the path degree of freedom. However, typically-used bulk polarization beam splitters (PBSs) are not the most ideal systems for this application, as the coupling from bulk to photonic chips leads to additional losses and unwanted complexity^8^. To overcome these issues, one can introduce on-chip beam splitting for the integration with waveguide-based SPDC sources. However, LiNbO_3_ waveguide-based beam splitters are typically designed for specific wavelengths such as asymmetric Y-junctions or heterogeneous directional couplers, resulting in a very narrow acceptable band^11–15^. The coherent tunneling adiabatic passage effect found in quantum physics^16^ has recently been optically realized in photonic waveguide systems, and a broadband beam coupling can be obtained in such counterintuitive adiabatic waveguide couplers^17,18^. Despite LiNbO_3_-based broadband adiabatic couplers designed for integration with type-0/type-I SPDC sources have been proposed and demonstrated^19,20^, it still remains a problem on designing both polarization- and wavelength-dependent beam splitters in a fully integrated manner for a type-II SPDC source. Another typical issue in type-II phase-matched SPDC lies in the temporal walk-off between signal and idler caused by the birefringence of the material. Hence one has to use a delay-line implemented by a second LiNbO_3_ crystal with the orthogonal crystalline orientation^7^ or a free-space Mach–Zehnder interferometer-type compensation setup^21^. A practical integrated type-II SPDC photon-pair generator will have to be stable, incorporate good control of polarizations in terms of both splitting ratios and mode sizes, generate photon pairs while filtering out the pump, minimize the signal/idler temporal walk-off, and have tunability through EO effect.

**Figure 1:**
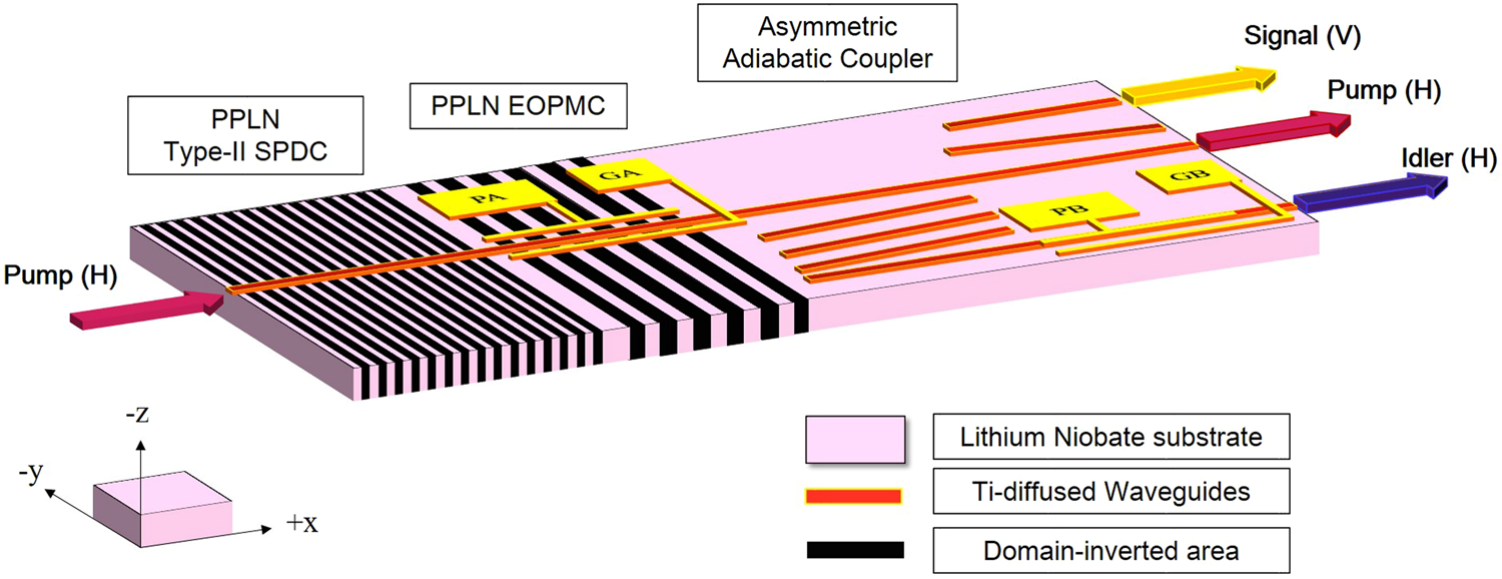
Schematic of a fully integrated quantum-polarization state source, with a Ti-diffused PPLN waveguide designed for type-II phase matching in the SPDC, an EO PMC with the electrodes PA and GA, and an AAC being used for photon-pair generation, EO-tunable polarization state conversion and polarization-wavelength beam splitting, respectively. The time-delay compensation of the cross-polarized modes can be completed by the electrodes PB and GB within the AAC.

In this work, we propose a concept of fully integrated quantum polarization state sources with three sections, i.e. photon-pair generation, polarization-mode conversion, and the pump filtering with time-delay compensation; as shown in Fig. 1. The first section realizes type-II SPDC, while the second section^2,3^, is used to modulate the polarization of photon-pairs by adjusting the applied electric-fields along crystal y-axis via the EO effect through the electrodes PA and GA. In the third section, pump filtering and quantum-polarization state preparation can be implemented via high-efficiency asymmetric adiabatic coupler (AAC) based on a specially designed waveguide array. The AAC device plays multiple roles of being a pump filter and a broadband PBS with a specific polarization splitting ratio depending on the wavelength, which allows to separate the V-polarized signals and the H-polarized idlers spatially, and can prepare different two-photon quantum-polarization states by taking the advantage of the wavelength-dependent splitting spectrum of the AAC. The temporal walk-off of two cross-polarization modes can be also compensated via the electrodes PB and GB by means of adjusting the applied electric-fields along crystal z-axis in an arm of AAC section. In this work, the third section is realized experimentally, while the first two stages have been shown in previous works and are simulated numerically within the framework of this paper.

## Results

### Design of the Asymmetric adiabatic coupler

**Figure 2:**
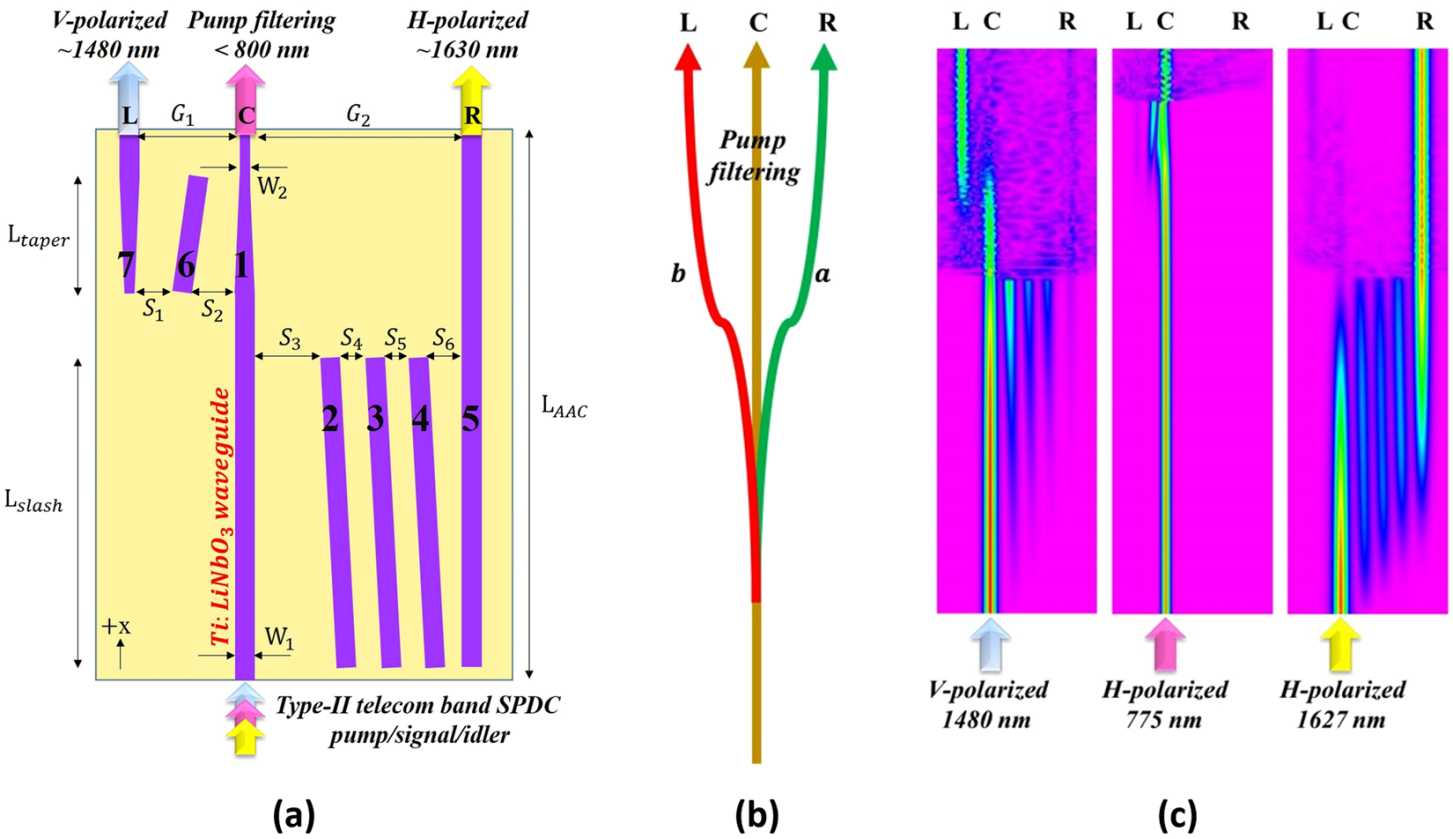
(**a**) The schematic diagram of the asymmetric adiabatic coupler, (**b**) the functional diagram of the AAC showing exits *L*, *C*, and *R* and paths *a* and *b* for SPDC pump filtering and signal/idler splitting, (**c**) the simulated power distributions in the AAC for a V-polarized 1480 nm, a H-polarized 775 nm, and a H-polarized 1627 nm inputs, respectively.

In general in the wavelength range of visible to near-IR, the refractive index change of *e*-wave mode (Δ*n*_*e*_) is about twice that of *o*-wave mode (Δ*n*_*o*_) for Ti-diffused waveguides made in a z-cut LiNbO_3_^22^, leading to obviously different coupling coefficients for the two cross-polarized modes. Using this feature, we integrated two adiabatic coupling structures of slash and taper types, which forms an AAC, to demonstrate the wavelength and polarization spatial mode splitter in the telecom band. Fig. 2a shows the design configuration of the AAC composed of 7 waveguides (No. 1 to 7) with a device length of L_AAC_. Here the slash and taper sections are with the length of L_slash_ and L_taper_, respectively. There are one input port and three output exits, with the exits marked as *L*, *C* and *R* in Fig. 2a and the functional diagram in Fig. 2b. Employing the different coupling conditions of two cross-polarized modes, we designed in a way that the first section (with slash waveguides) of the AAC maintains 1480-nm V-polarized signals and 775-nm H-polarized pump in waveguide 1 and in the meanwhile maximizes the power of 1627-nm H-polarized idlers transferred to waveguide 5, which then leaves the device from exit *R*. The use of three slash waveguides here rather than a single slash waveguide that we have employed for working on a specific polarization mode^20^ is the key to prevent the power transfer of the V-polarized (TM-polarized) mode while permitting the adiabatic coupling of the H-polarized (TE-polarized) mode from waveguide 1 to waveguide 5. No waveguide configuration in LiNbO_3_ could be found in this spectral region for the adiabatic light transfer of the V-polarized mode without the occurrence of the power coupling of the H-polarized mode (from waveguide 1 to waveguide 5) due to the polarization-dependent mode characteristics (dispersion, size, etc.) in Ti:LiNbO_3_ waveguides. The major reason to use the taper waveguides in the downstream section of the AAC for adiabatic coupling of the V-polarized mode from waveguide 1 to waveguide 7 (then to exit *L*) is to effectively shorten the coupler length; see the analysis made in Methods. In the ideal case, we can assume that the propagation constants of slash waveguides 2 to 4 are the same (*β*_2_ = *β*_3_ = *β*_4_ = *β*_*sl*_), and the propagation constant of straight waveguides 1 and 5 are the same (*β*_1_ = *β*_5_ = *β*_*s*_). By engineering accessible geometric parameters of waveguides, one can tailor the adiabaticity and therefore the splitting ratio spectrum that depends both on frequency and polarization. We utilize the Beam Propagation Method^23^ to simulate the output performance and confirm the optimal parameters of the AAC. The AAC parameters used in this work are *L*_*AAC*_ = 51 *mm*, *L*_*slash*_ = 30 *mm*, *L*_*taper*_ = 15 *mm*, *S*_1_ = 3 *μm*, *S*_2_ = 8 *μm*, *S*_3_ = 11 *μm*, *S*_4_ = 10 *μm*, *S*_5_ = 10 *μm*, *S*_6_ = 14 *μm*, *G*_1_ = 18 *μm*, *G*_2_ = 67 *μm*, *W*_1_ = 7 *μm*, *W*_2_ = 5 *μm*. The simulated evolution of the light transfer during propagation of three representative input waves are in the AAC shown in Fig. 2c.

### Characterization of the AAC

**Figure 3:**
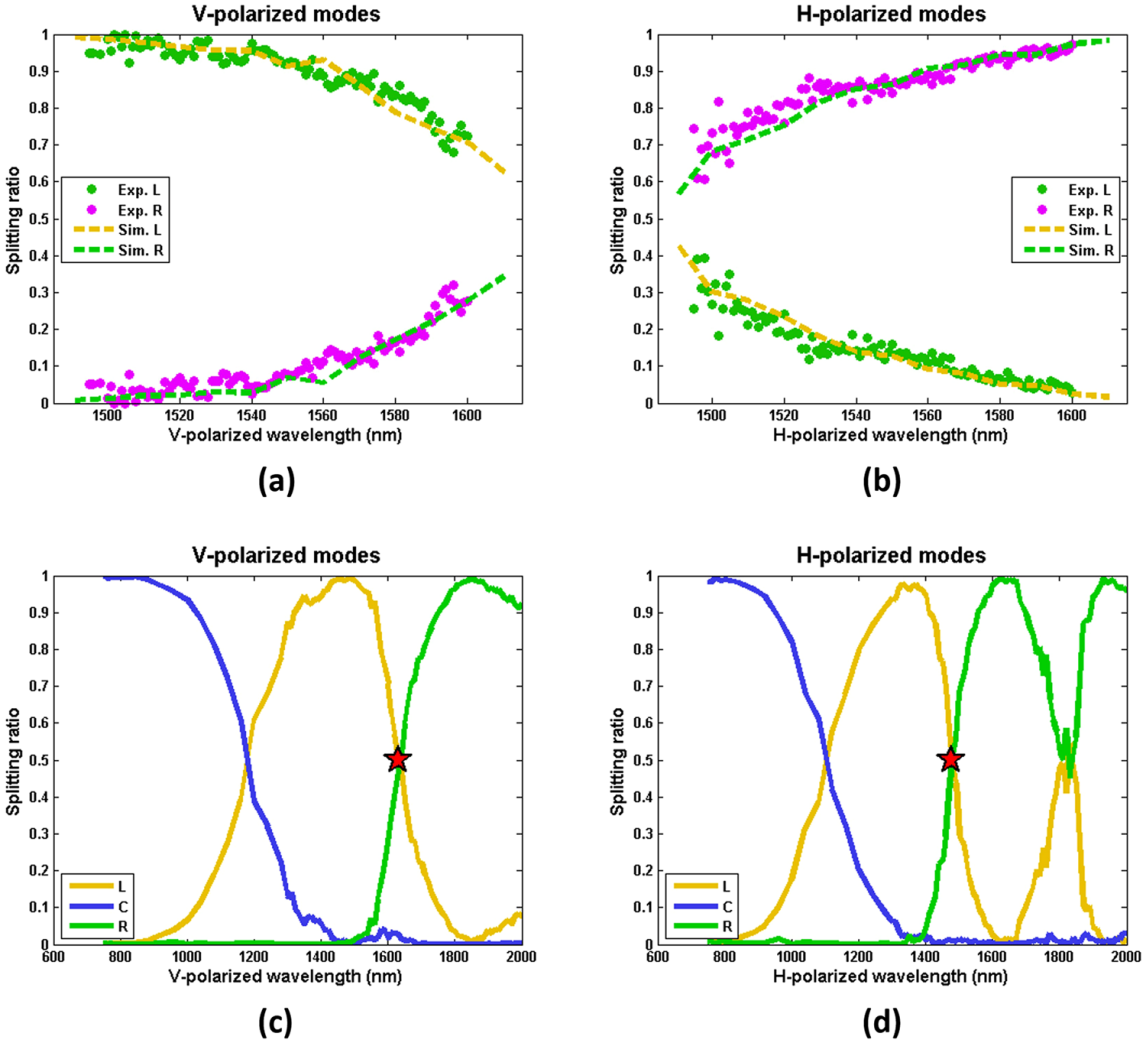
Measured and simulated power splitting ratios of the AAC for (**a**) V-polarized and (**b**) H-polarized fundamental modes over a wavelength range from 1495 nm to 1600 nm, where the results from exits *L* and *R* are shown as green and purple dots for experimental measurements and gold and green dash curves for simulation results, respectively. (**c**,**d**) Simulated splitting ratios of the AAC for V- and H-polarized fundamental modes, respectively, for a wavelength range from 750 nm to 2000 nm, where the red stars mark 50%/50% splitting ratios on two special wavelengths at V-polarized 1631 nm and H-polarized 1477 nm, exactly a photon pair generated via a type-II SPDC when pumped by an H-polarized 775 nm laser, upon which the AAC can act as a non-polarizing beam splitter for quantum-polarization state generation.

The polarization- and frequency-dependent splitting ratios of the AAC are characterized by the wavelength from 1495 nm to 1600 nm of a CW laser source, with the results plotted in Fig. 3a and b. The experimental results (dots) are compared to the simulation results (dashed curves). For a broader range of wavelength, we show the simulated splitting spectra of the same AAC for both vertically (V-) polarized and horizontally (H-) polarized fundamental modes in the wavelength range from 750 to 2000 nm [see Fig. 3c and d], respectively.

As seen in Fig. 3, the AAC splits H- and V- polarized modes with the different ratio for different wavelengths, where the experimental results are highly consistent with predictions given by simulation. The bandwidths of the −17 dB extinction/splitting ratio of the AAC for the H- and V-polarized modes are 60 and 70 nm, respectively. In Fig. 3c and d, the red stars mark the wavelengths (1631 and 1477 nm) where the V- and H- modes are equally split to the two exits. Interestingly, these points can be utilized as a 50/50 non-polarizing beam splitter for photon pairs generated via type-II SPDC (here with V-polarized 1631 nm signal and H-polarized 1477 nm idler being the photon pair that is generated by the H-polarized 775 nm pump), where the splitting ratios of the signal and idler are both 0.5 at exits *L* and *R*.

### Manifestation of the AAC in quantum-polarization state preparation

The non-trivial dependence of the splitting ratio of the AAC on polarization and wavelength allows for practical applications in controlling the quantum-polarization states. For photons generated via type-II phase-matched waveguides, signal and idler photons are in pairs with orthogonal polarizations under energy and momentum conservation. If such photons are launched into an arbitrary beam splitter with exit paths ***a*** and ***b***, one can end up with a state described by

1 $$|{\rm{\Psi }}\rangle =\alpha ({|H\rangle }_{a}{|V\rangle }_{b})+\beta (|V{\rangle }_{a}|H{\rangle }_{b})+\gamma (|H{\rangle }_{a}|V{\rangle }_{a})+\zeta (|V{\rangle }_{b}|H{\rangle }_{b}),$$

where ${|\alpha |}^{2}+{|\beta |}^{2}+{|\gamma |}^{2}+{|\zeta |}^{2}=1$. The last two terms in Eq. (1) describe the case when both signal and idler photons are going to the same port, which is not recognizable by on-off detectors. For our polarization- and wavelength-dependent AAC, here we focus on the cross-correlation between paths a and b, where we can define a preparation efficiency as $\eta =|\alpha {|}^{2}+|\beta {|}^{2}$.

**Figure 4:**
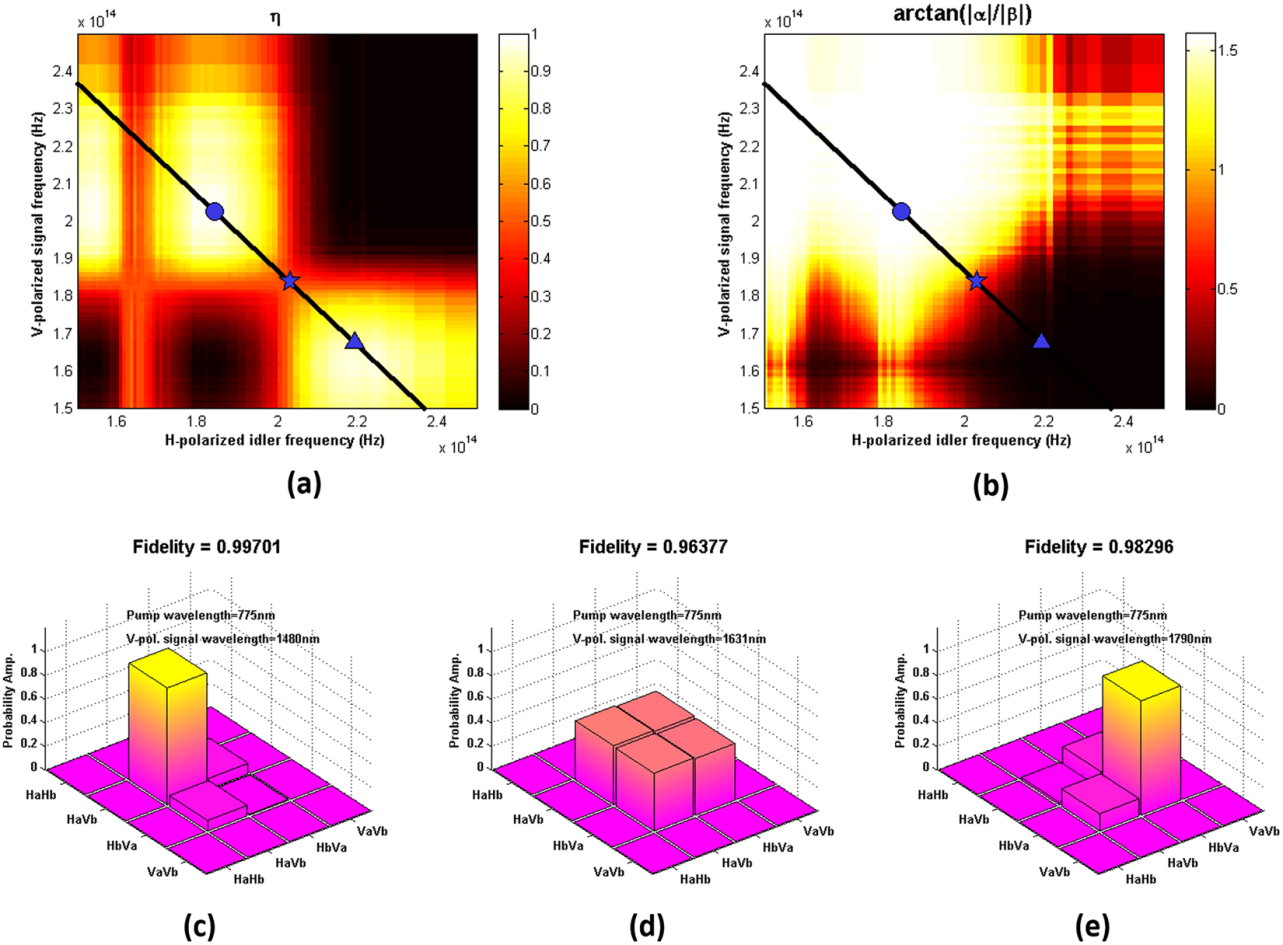
(**a**) The simulated quantum polarization state preparation efficiency and (**b**) the intensity map of ${atan}(|\alpha |/|\beta |))$ of quantum polarization-path states of ${\rm{\Psi }}=\alpha {(|H\rangle }_{a}|V{\rangle }_{b})+\beta {(|V\rangle }_{a}|H{\rangle }_{b}))$ in frequency domain with blue star markers denoting the signal and idler photon-pair wavelengths at 1477 nm (H-polarized mode) and 1631 nm (V-polarized mode), respectively, in which the type-II SPDC source can approach to a pure quantum polarization-path entangled state (see (**d**)) when working with the proposed AAC. (**c**,**d**), and (**e**) are the simulated density matrices for a 775-nm pumped QPM type-II SPDC photon-pair source when working with the proposed AAC for phase-matched V-polarized signals at 1480, 1631, and 1790 nm (corresponding idlers at 1627, 1477, and 1367 nm), respectively.

For quantum-polarization state preparation, we can obtain different combination of wavelengths of generated photon pairs from the SPDC waveguide via varying the pump wavelength or changing the phase-matching condition via varying the temperature. When such a photon pair passes through our polarization- and wavelength- dependent AAC, the variable wavelengths of photon pairs will lead to variable polarization-path states due to different splitting ratios; hence the final quantum-polarization state is controllable by varying the temperature or pump wavelength. For instance, the black lines in Fig. 4a and b are the type-II phase-matched SPDC photon-pair tuning curves under a H-polarized 775 nm pump. Here we assumes the AAC works at a temperature of 25 °C, but the temperature of the SPDC waveguide can be independently controlled. Hence we can reach different positions along the black line by varying the temperature of the SPDC waveguide, where the quantum-polarization state coming out of the AAC will change accordingly. Figure 4c–e show the simulated density matrices for the SPDC source when working with the proposed AAC and operated at three phase-matched conditions for the V-polarized signal wavelengths at 1480 nm, 1631 nm, and 1790 nm, corresponding to those positions marked by blue circle, star, and triangle in Fig. 4a and b, respectively. In general, the parameters *α*, *β*, *γ*, *ζ* are complex, however, since the measured output power and the simulated fitting curves for power splitting efficiencies only gives information of absolute values, we plot the density matrices of the quantum-polarization state preparation efficiency and the arctangent values of ratio $|\alpha |/|\beta |$ in absolute values. The results shown in Fig. 4c–e imply one can control/tune the quantum states by simply tuning the phase-matching condition (along the black lines in Fig. 4a and b over a broad band) of the SPDC under the same pump wavelength without installing extra components or modulation mechanisms. The simulation result shown in Fig. 4d suggests a unique working condition in which the type-II SPDC source can be possible to generate a Bell’s state (a polarization-path entangled state with the fidelity of 0.96377) when working with the proposed AAC.

## Discussion

Unlike narrow-band (MHz) photon pair generations via atomic^24^ and atom-like^25^ media with *χ* ^(3)^ (or *χ* ^(3)^:*χ* ^(3)^) nonlinearity, this work focuses on the application of the *χ* ^(2)^ SPDC sources to integrated optical circuits over a broader band (THz). In terms of the conversion bandwidth, type-II phase-matched waveguide SPDC sources offer an important advantage of having high-brightness output in a much narrower spectrum in comparison with type-0 phase-matched photon-pair sources^6–8,21^. Besides, in type-II sources, when working in the path-entanglement scheme, the photon-pair sources can be transformed to the polarization entanglement via the polarization-dependent splitting setup, which is difficult to achieve for type-0 photon-pair sources with the same polarization of signal and idler modes. Since the polarization crosstalk of Ti-diffused LiNbO_3_ waveguides is very limited (−20 to −25 dB typically)^14^, the good polarization maintaining of the waveguides makes such an integrated setup suitable for implementing the quantum polarization preparation devices.

For practical applications, the birefringence-induced time delay of photon pairs can be compensated by simply adjusting the relative phase of the H- and V-polarized modes with the electric field applied along the crystal z-axis in an arm of the AAC section (see Fig. 1)^7^. According to our previous characterization on a type-II, degenerated SPDC source in Ti-diffused PPLN waveguides, the PPLN grating periods for generating photon pairs at 1530–1600 nm are 8.8 ~ 9.7 *μ*m for working temperature at 120 °C. The spectral bandwidth is ~3.66 nm-cm and the temperature tuning rate is *dλ*/*dT* ~ −0.18 *nm*/°C in this working range. On the other hand, based on our previous study, we estimate the spectral bandwidth of an EO PMC built in Ti-diffused PPLN waveguides is around 5 nm for a 6-mm device length at 1550-nm band^26^. The temperature tuning rate of the PPLN EO PMC is *dλ*/*dT* ~ −0.8 *nm*/°C. The AAC is characterized by broad spectral and temperature bandwidths due to the nature of the adiabatic light transfer process. To deal with the possible different drifts of the working temperature from the design value (25 °C in this work) of each device in the fully integrated quantum circuits (see Fig. 1) due to the different fabrication errors or/and nonuniform temperature control of the respective devices, we can, for instance, adopt a multiple grating PPLN structure^26^ covering a suitable range of grating periods (corresponding to a range of phase-matching temperatures) with respect to the design value in either the SPDC or the EO PMC section. Another interesting approach to try would be to use the reverse adiabatic coupling, which may be more efficient and robust in comparison with the counter-intuitive coupling scheme in LiNbO_3_ waveguides^27^.

## Methods

The energy coupling of modes in the AAC can be described by the coupled-mode equations and simulated by the Beam Propagation Method^5^. For the first section of the AAC, it consists of three identical slash waveguides sandwiched by two identical straight waveguides (see Fig. 2a). The field of the modes propagating along the *x* axis in the *n-th* waveguide can be expressed as ${a}_{n}(x)={b}_{n}(x)\exp (i\beta x)$ where *b*(x) is the mode amplitude and *β* is the propagation constant. The phase difference between the slash and straight waveguide modes is ${\rm{\Delta }}{\beta }_{s}={\beta }_{sl}-{\beta }_{s}$, and the coupling coefficients of neighboring waveguides are ${K}_{n,n+1}(x)={K}_{n+\mathrm{1,}n}(x)$. The power transfer behavior of such a slash adiabatic coupling system can then be simulated by solving the coupled-mode equations given by $-id{\bf{b}}(x)/dx={\bf{M}}(x){\bf{b}}(x)$, where

2 $${\bf{M}}(x)=[\begin{array}{ccccc}0 & {K}_{1,2}(x) & 0 & 0 & 0\\ {K}_{2,1}(x) & {\rm{\Delta }}{\beta }_{s} & {K}_{2,3} & 0 & 0\\ 0 & {K}_{3,2} & {\rm{\Delta }}{\beta }_{s} & {K}_{3,4} & 0\\ 0 & 0 & {K}_{4,3} & {\rm{\Delta }}{\beta }_{s} & {K}_{4,5}(x)\\ 0 & 0 & 0 & {K}_{5,4}(x) & 0\end{array}]$$

In design, we found the first (slash adiabatic coupling) section of the AAC has to be relatively long to enhance the adiabaticity of the system to permit only the power transfer of the H-polarized mode from waveguide 1 to waveguide 5. To reduce the total device length, we adopt a taper structure in waveguides 1 and 7 as a “shortcut to adiabaticity” design^28^ in the second section of the AAC to minimize the adiabatic coupling length to transfer the power of V-polarized signals to the waveguide 7 and remain the H-polarized pump in waveguide 1. This taper design is not applied to the first section because it will lead the H-polarized mode to have a high propagation loss. Similar to the above analysis, we can express the *x*-dependent propagation constants of the waveguides 1 and 7 in this section by ${\beta }_{1}(x)={\beta }_{t}-x\delta \beta$ and ${\beta }_{7}(x)={\beta }_{t}+(x-{L}_{taper})\delta \beta$, respectively, where *δβ* is the gradient rate of the propagation constant of the taper waveguides, and ${\beta }_{t}\equiv {\beta }_{7}(x={L}_{taper})={\beta }_{1}(x={0}_{taper})$. Accordingly, the transfer matrix for this taper adiabatic coupling system can be written as:

3 $${\bf{M}}(x)=[\begin{array}{ccc}-x\delta \beta  & {K}_{1,6}(x) & 0\\ {K}_{1,6}(x) & {\rm{\Delta }}{\beta }_{t} & {K}_{6,7}(x)\\ 0 & {K}_{6,7}(x) & (x-{L}_{taper})\delta \beta \end{array}],$$

where *K*_*a*,*b*_(*x*) is the coupling coefficient between waveguides *a* and *b* and Δ*β*_*t*_ = β_6_ − *β*_*t*_.

The waveguide devices were fabricated using the titanium thermal diffusion (TTD) method^20^. For the AAC device, waveguide system was fabricated in a 51-mm long, 10-mm wide and 0.5-mm thick z-cut LiNbO_3_ crystal. First, Ti strips of a thickness of 90 nm and a layout as schematically shown in Fig. 2a was first coated on the −z surface of the crystal using the standard lithographic and lift-off processes. The titanium diffusion processes is then performed in a high-temperature furnace at 1035 °C with a constant oxygen flow for 12 hours. The waveguides support the single (fundamental) mode guiding for both TM and TE modes in the spectral range 1400–1700 nm. The performance of the waveguide device was characterized by using an external cavity laser (ECL) tunable from 1495 to 1640 nm. The output of the ECL was polarization controlled and maintained before it was butt coupled into the waveguides. The waveguide sample was mounted on a multi-axis precision translation stage. The measured propagation losses of Ti-diffused LiNbO_3_ waveguides are ~0.3 and ~0.6 dB/cm for V-polarized and H-polarized 1550 nm modes, respectively. In addition, we proposed in this work a fully integrated quantum optical circuits as Fig. 1 shown. Besides the waveguide fabrication, the realization of this on-chip quantum polarization-path-dependent light source involves the domain inversion process in fabricating the PPLN SPDC and the PPLN EO PMC. The main fabrication process for Ti-diffused PPLN waveguides includes lithographic Ti strips definition, thermal in-diffusion, surface polish, and electric field poling^29^. Optical polish on the +z surface is required before the crystal poling to remove the domain-inverted layer on the +z surface of the crystal formed during the high-temperature TTD process^30^. After the waveguide and PPLN fabrication, the coating of the stripe electrodes (Ti/Au) along the waveguide sides in the PPLN PMC section and over the waveguides buffered by an oxide (SiO_2_) layer in one arm of the AAC section will be applied for relevant EO modulations accessing the EO coefficients *r*_51_ and *r*_33_, respectively. The device fabrication is then accomplished after performing an optical polish on the end faces of the crystal. In addition, one can use the end-fiber pigtailing, housing, and packaging techniques to facilitate the integration of the LiNbO_3_ sample with the fiber-optical systems or quantum source modules.
